# Antimicrobial Resistance and Pathogenicity of *Aliarcobacter butzleri* Isolated from Poultry Meat

**DOI:** 10.3390/antibiotics12020282

**Published:** 2023-02-01

**Authors:** Maria Gabriela Xavier de Oliveira, Marcos Paulo Vieira Cunha, Luisa Zanolli Moreno, André Becker Simões Saidenberg, Mônica Aparecida Midolli Vieira, Tânia Aparecida Tardelli Gomes, Andrea Micke Moreno, Terezinha Knöbl

**Affiliations:** 1 Faculdade de Medicina Veterinária e Zootecnia, Universidade de São Paulo, USP, Butantã, São Paulo 05508-270, SP, Brazil; 2 Departamento de Microbiologia, Imunologia e Parasitologia, Escola Paulista de Medicina–Universidade Federal Paulista UNIFESP, São Paulo 04023-062, SP, Brazil

**Keywords:** *Aliarcobacteriosis*, multidrug resistance, food safety

## Abstract

*Aliarcobacter butzleri* (*A. butzleri*) is an emergent zoonotic food-related pathogen that can be transmitted through the consumption of poultry meat. Data regarding the pathogenicity and resistance of *A. butzleri* are still scarce, and the presence of virulent MDR strains of this zoonotic pathogen in poultry meat is an issue of particular concern to public health. This study aimed to characterize the pathogenicity and antimicrobial resistance profiles of *A. butzleri* strains isolated from poultry meat sold at retail markets in São Paulo, Brazil. The minimum inhibitory concentrations of 27 strains were determined using the broth microdilution method. The results showed that 77.7% of the isolates were resistant to clindamycin, 62.9% to florfenicol, 59.2% to nalidixic acid, 11.1% to azithromycin, 7.4% to ciprofloxacin and telithromycin, and 3.7% to erythromycin and tetracycline, although all were susceptible to gentamicin. Moreover, 55.5% of the virulent isolates were also multidrug-resistant (MDR). Three strains were selected for pathogenicity tests in vitro and in vivo. The tested strains expressed weak/moderate biofilm production and showed a diffuse adhesion pattern (3 h) in HeLa cells and toxicity in Vero cells (24 h). Experimental inoculation in 11-week-old chicks induced a transitory inflammatory enteritis. Intestinal hemorrhage and destruction of the intestinal crypts were observed in the rabbit ileal loop test. Considering the fact that Brazil is a major exporter of poultry meat, the data from this study point to the need of improvement of the diagnostic tools, as well as of the adoption of surveillance guidelines and more specific control strategies to ensure food safety, reducing the presence of pathogenic MDR strains in broilers.

## 1. Introduction

The genus *Aliarcobacter* is a member of the Campylobacteraceae family, and infection with the species *A. butzleri* is associated with abdominal cramps, gastroenteritis, severe diarrhea, blood in stools, and sepsis in humans, constituting serious hazards to human health [1,2]. Infection with these bacteria is commonly related to the ingestion of contaminated water, vegetables, seafood, meat, raw milk, and dairy products [3,4,5,6,7,8,9]. However, the main cause of food-born outbreaks has been attributed to the contamination of poultry meat by *A. butzleri* [6,10,11].

Arlicobacteriosis is a worldwide disease, and although there are few reports of human infection, the disease has already been described in several European countries, such as Italy, Spain, French, United Kingdom, Germany, Belgium, Denmark, as well South Africa, Japan, Thailand, and Hong Kong [1,12]. In Latin America, *A. butzleri* has been isolated from humans, animals, and food sources, mainly in Chile and Brazil [5,6,13].

The real incidence rate of *A. butzleri* is probably underestimated due the lack of diagnostics and misidentification, but some reports point this species as the fourth most frequent among *Campylobacter*-like isolates from human stool [2,12]. Besides that, the prevalence of MDR strains of *Aliarcobacter* is much higher than that of *Campylobacter* [14,15]. According to Son et al. [14], the resistance levels of *A. butzleri* range from 72.9 to 80.9%, and high rates of resistance to penicillins, aminopenicillins (ampicillin), and cephalosporins have been reported in bacterial isolates from humans and companion and production animals [14]. 

The pathogenicity of *Aliarcobacter* is still poorly understood, but the ability of some species to produce inflammatory reactions, enteritis, and septicemia have been investigated in vitro (cell cultures) and in vivo (laboratory animals) [1]. *A. buztleri* binds and invade Caco-2 cells and promotes cytotoxic effects on Vero cells. In vivo, *A. buztleri* may colonize the liver, kidney, ileum, and brain of rodents, which develop diarrhea and changes in hematological parameters, which are dose-dependent [16]. 

Nine putative virulence genes have been employed in molecular approaches for the characterization of the potentially pathogenic species of *Aliarcobacter*: *cadF* (fibronectin protein), *ciaB* (invasion), *cj1349* (fibronectin protein), *pldA* (lysis of erythrocytes), *mviN* (peptidoglycan biosynthesis), *tlyA* (hemolysin), *hecB* (hemolysin protein activation), *hecA* (hemagglutinin filaments, which are involved in attack, aggregation, and cell death), and *irgA* (regulator of protein for iron acquisition) [6,16]. In a previous study, we identified *A. butzleri* in 11.66% of the poultry meat marketed in São Paulo, Brazil, and 100% of the strains were positive for *ciaB, cj1349, mviN.* The prevalence of *pldA, tlyA*, and *cadF* was 97.2%, 94.2%, and 74.2%, respectively. The prevalence of the genes *hecA, irgA*, and *hecB* ranged between 40 and 45.7% [6]. Brazil is a major global exporter of broilers. Studies focusing on the antimicrobial susceptibility profiles of *Aliarcobacter* spp. in chicken meat are rare, even though the presence of virulent and MDR strains of these bacteria represents an important public health issue [6]. Moreover, the different methodologies employed to determine the susceptibility to antimicrobials have impeded the interpretation and effective comparison of rates of resistance among different countries [1]. The classical disk diffusion method for assessing antimicrobial resistance is simple and commonly used, but the diameters of the inhibition zones are often difficult to discriminate with precision. In contrast, the broth microdilution method enables minimum inhibitory concentration (MIC) values to be determined accurately and reproducibly, and a standard protocol for antimicrobial susceptibility testing of *Aliarcobacter* spp. using this method has been proposed [15]. 

In light of the above, the present study aimed to establish the antimicrobial resistance profiles of *A. butzleri* strains isolated from poultry retail meat sold in markets in São Paulo city (Brazil), by determining the MIC values using the broth microdilution method. Additionally, three strains were selected for the analysis of pathogenicity in vitro and in vivo. 

## 2. Results

The MIC values obtained for the 27 *A. butzleri* isolates revealed that 22 showed resistance to at least one antimicrobial, with 77.2% (21/27) being resistant to clindamycin, 62.9% (17/27) to florfenicol, 59.2% to nalidixic acid (16/27), 11.1% (3/27) to azithromycin, 7.4% (2/27) to ciprofloxacin, and 3.7% (1/27) to erythromycin, telithromycin, and tetracycline (Table 1). All the tested isolates were susceptible to gentamicin. The results of MIC_50_ and MIC_90_ are presented in Supplementary Material Table S1. 

Ten different antimicrobial resistance profiles (R1–R10) were identified among the isolates of *A. butzleri* (Table 1). The antimicrobial resistance profiles revealed that 55.5% (15/27) of the virulent isolates of *Aliarcobacter* were MDR (profiles R6 to R10, Table 1).

**Figure 1:**
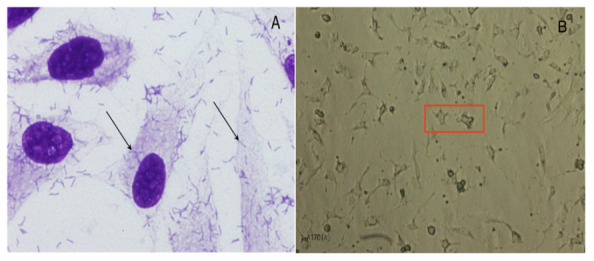
(**A**) Adherence of *Arcobacter butzleri* to HeLa cells. (**B**) VERO cells after the addition of *A. butzleri* supernatant, presenting elongated cells with rounded vacuoles (Red box).

The phenotypical tests of AB165 and AB170 showed a weak biofilm formation, with OD of 0.695 ± 0.055 and 0.679 ± 0.143, respectively. However, these strains presented moderate adherence to HeLa cells in 3 h. (Figure 1A). The strain AB167 presented a moderate biofilm formation, with OD of 0.926 ± 0.027, but did not adhere to HeLa cells in 3 h. The OD of the positive control, enteroaggregative *E. coli* 042, was 1.002 ± 0.076.

All strains were positive in the toxicity tests, with observable effects in the form of vacuolation and destruction of Vero cells (Figure 1B). 

**Figure 2:**
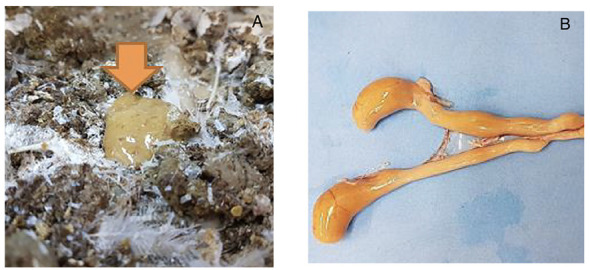
Feces and cecum of birds, four days after the inoculation of *A. butzleri* AB170 strain. (**A**) The orange arrow shows the diarrheagenic aspect of poultry feces. (**B**) Bird’s ceca with an orange content.

The chicken inoculation of *A. butzleri* promoted a mild diarrhea (Figure 2A) between the second and the fourth days after the challenge), with the presence of an orange content and gas in the cecum (Figure 2B) and hyperemia of the intestine. The strains were recovered from feces on the 2nd^.^, 3rd,^.^ and 4th^.^ day after the challenge, but the culture was negative after the 5th^.^ day. These changes were not observed in birds of the non-challenged group. 

**Figure 3:**
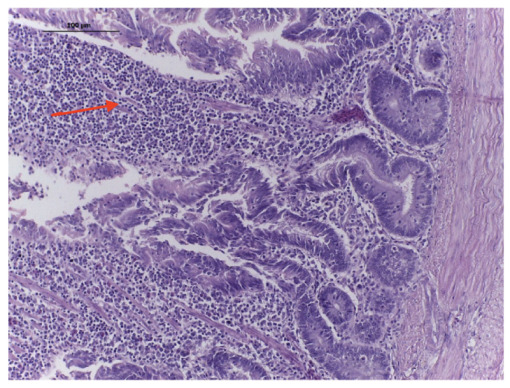
Micrograph of a chicken intestine, four days after the inoculation of *A. butzleri* AB170 strain, presenting inflammatory infiltrate cells (Red arrow). HE × 100.

The microscopic changes showed a moderate inflammation of the intestinal crypts, with focal dilation and thickening of the villi and inflammatory infiltrate with lymphocytes, heterophils, plasmocytes, and macrophages in the lamina propria (Figure 3). Flattening of villi, with a decrease in length and tissue destruction, was also observed, as well as a slight increase in mitosis figures in the crypt. In the lumen, there were multiple bacterial colonies (bacilli).

**Figure 4:**
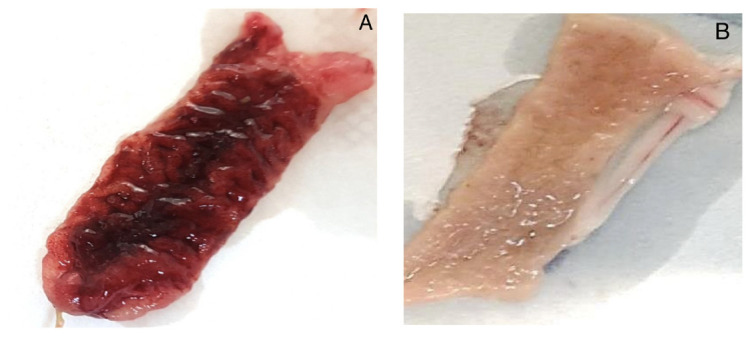
Macroscopic evaluation of rabbit ileal loops, 12 h after the inoculation of the AB170 strain. (**A**) Macroscopic hemorrhagic aspect of the intestine 12 h after the inoculation of *A. butzleri*. (**B**) Negative control. Intestinal tissue inoculated with PBS.

**Figure 5:**
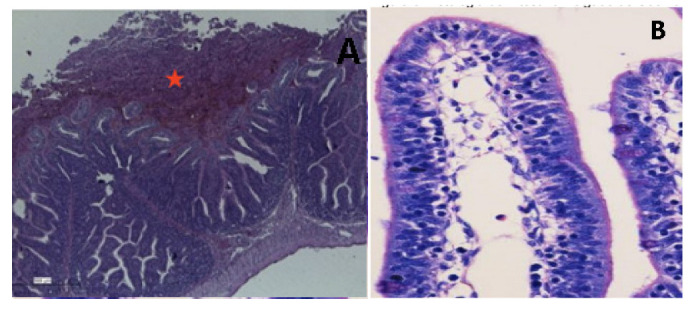
Ileal histopathology of an experimentally inoculated rabbit, 12 h after the inoculation of the AB170 strain. (**A**) Ileal loop showing erythrocytes and debris in the lumen (Red star). HE × 100. (**B**) Negative control. Intestinal tissue is preserved, HE × 200.

**Figure 6:**
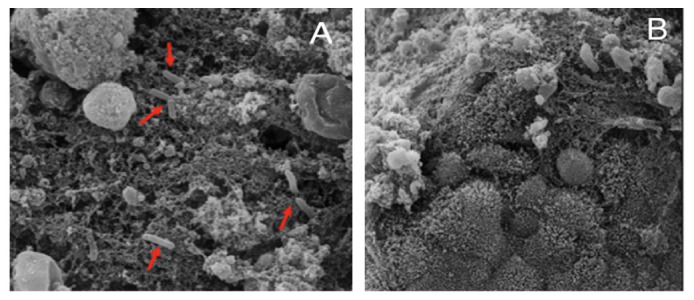
Scanning Electron Microscopy (SEM) of the small intestine in the inoculated rabbit ileal loops model, 12 h post inoculation of the AB170 strain. (**A**) Destroyed villi and presence of bacilli in the extracellular matrix (Red arrows). (**B**) Negative control. Intestinal crypts are preserved.

The rabbit ileal loops model showed that the strains AB165, AB167, and AB170 induced intestinal hemorrhage (Figure 4A),12 hours after the inoculation. The microscopic analysis of these loops showed the presence of erythrocytes and cell debris in the lumen (Figure 5A). There were some submucosal changes, with discrete multifocal ectasia of the lymphatic vessels. Scanning electron microscopy showed the presence of some bacilli in the extracellular matrix, and the villi were destroyed (Figure 6A). None of these changes were observed in the negative control (Figure 4B, Figure 5B, Figure 6B). 

## 3. Discussion

The unrestricted use of antibiotics in both human and animal populations has contributed to a global increase in infections by MDR pathogens. However, information on the antimicrobial resistance patterns of *A. butzleri*. is still limited [17,18,19,20,21]. In addition, the lack of standardization in the methodologies employed and in the interpretation of the applied criteria has generated challenges in analyzing the resistance data, resulting in misleading comparisons [1,15]. 

According to the antimicrobial susceptibility data reported herein, the studied *Aliarcobacter* isolates showed the highest resistance percentage (77.2%) to the lincosamide class of antibiotics (clindamycin) and a slightly lower resistance percentage to phenicol (florfenicol) and quinolone (nalidixic acid) antibiotics, corresponding to 62.9% and 59.2%, respectively. A percentage (81.5%; 22/27) of *A. butzleri* isolates were resistant to at least one antimicrobial, and a high percentage (55.5%) were MDR. 

Treatment of aliarcobacteriosis with a course of antibiotics is recommended for patients presenting severe clinical signs but not for those with only mild symptoms [22]. Since different species of the genus *Aliarcobacter* are closely related to those of *Campylobacter*, the drugs of choice are fluoroquinolones (such as ciprofloxacin and enrofloxacin), macrolides, and, less commonly, tetracyclines and aminoglycosides [23]. In a study involving 174 isolates of *Aliarcobacter* obtained from broiler carcasses collected from a poultry processing plant in the United States, Son et al. [14] found that the *A. butzleri* isolates were highly resistant to clindamycin (90%), followed by azithromycin (81.4%) and nalidixic acid (23.6%). Our results also point to clindamycin as the least effective drug, with 77.2% of resistant strains, although the 59.2% resistance to nalidixic acid reported herein exceeds that established in the American study, while the resistance percentage to azithromycin (7.5%) is substantially lower. 

According to Van den Abeele et al. [24], 13% of the *A. butzleri* strains isolated from Belgian patients with gastroenteritis showed resistance to ciprofloxacin, with MIC_90_ values >32 mg/L. Furthermore, Shah et al. [25] reported that strains of *A. butzleri* were resistant to ampicillin (56%), cefotaxime (33%), and ciprofloxacin (33%) but susceptible to gentamicin and enrofloxacin. In contrast, our data revealed that only 7.4% of *Aliarcobacter* isolates from broiler meat were resistant to ciprofloxacin, with MIC_90_ values of 0.5 µg/mL (Table S1). Unlike other countries, Brazil has never employed fluoroquinolones as growth promoters in poultry, limiting their use to the therapeutic treatment of specific diseases, for example, paratyphoid *Salmonella*. This restriction may account for the epidemiological differences found between the data obtained in the present study and those of reports from other countries. 

There is evidence to support the hypothesis that resistance patterns in production animals are similar to those found in human isolates, and, for this reason, resistance to fluoroquinolones is of significant concern, since drugs of this class are used in therapies for both humans and animals. In this context, Van Boven et al. [26] collected cloacal swabs from broilers that had received enrofloxacin and observed that this treatment led to the rapid selection of resistant isolates of *Campylobacter jejuni,* all of which exhibited high frequencies of mutations in the *gyr*A gene. Interestingly, Van den Abeele et al. [24] carried out genomic analyses of ciprofloxacin-resistant strains of *A. cryaerophilus* isolated from stool samples of patients with gastroenteritis and established that all carried a mutation at position 254 of *gyr*A, thereby pointing to a mechanism of acquired resistance. In a recent study of *A. butzleri* strains isolated from a variety of animal, vegetable, dairy, and aquatic sources, Isidro et al. [9] confirmed that all ciprofloxacin- and levofloxacin-resistant isolates presented the same mutation in *gyr*A. These authors also reported the presence of the *blaOXA-15*-like gene in the strains [9]. 

Although the present study showed that the isolates of *A. butzleri* from poultry meat were sensitive to ciprofloxacin, a high resistance rate (62.9%) was recorded to florfenicol. This finding can probably be attributed to the use in Brazil of this antimicrobial for the prevention of respiratory diseases during the early stages of poultry breeding, thereby generating a selection pressure for resistant strains in the intestinal microbiome. To the best of our knowledge, no information is currently available that would allow a country-by-country comparison of the rates of resistance of *Aliarcobacter* spp. to florfenicol. 

It is important to note that the use of macrolides for the management of avian mycoplasmosis can also result in the selection of resistant strains in the family Campylobacteraceae [17]. For example, Logue et al. [27] reported that the administration of tylosin to turkeys over a four-week period gave rise to an increase in macrolide-resistant strains of *Campylobacter* detected at slaughter. However, the results from the present study demonstrate that the rates of resistance to azithromycin, erythromycin, and telithromycin in broilers produced in Brazil are low (11.1, 3.7, and 3.7%, respectively). 

Gentamicin and tetracycline are also considered effective drugs for the treatment of *Aliarcobacter* infections [28,29]. Van den Abeele et al. [24] evaluated the susceptibility of strains of *A. butzleri* isolated from human patients to these drugs and found that tetracycline presented the highest clinical efficiency. In addition, Isidro et al. [9] reported that all the studied strains of *A. butzleri* were susceptible to gentamicin. These findings agree with the data obtained in the present study, whereby the level of resistance of *Aliarcobacter* isolates to tetracycline was low (3.7%), and no strains exhibited resistance to gentamicin. According to Isidro et al. [9], the resistance of *A. butzleri* to tetracyclines is likely associated with the inactivation of a *tet*R gene repressor [9]. The resistance mechanisms of *Aliarcobacter* spp. are usually of a chromosomal nature, for example, related to mobilizable chromosomal genomic islands [30,31]. Isidro et al. [9] performed a comparative genomic study of 49 strains of *A. butzleri* and reported the presence of an array of efflux pump-related genes, some of which were associated with drug extrusion. 

In our study, 10 different resistance profiles (R1–R10) were identified among the 27 isolates of *A. butzleri* (Table 1). Five of the isolates (18.5%) showed sensitivity to all antimicrobials tested (profile R1), while 11 isolates (40.7%) were resistant to clindamycin, nalidixic acid, and florfenicol (profile R6) (Table 1). Our findings differ from those of Son et al. [14], who found that 16.1% (28/174) of the strains tested presented a single MDR profile involving resistance to azithromycin, nalidixic acid, and clindamycin (profile R8 in the present study). 

In addition to the multiple resistance profiles of *A. butzleri*, there is a concern about the pathogenicity of the agent. However, few experimental models have been employed to verify the risks associated with infections of humans and bird.. To assess this information, we selected three virulent strains for in vitro and in vivo assays. 

Our study confirmed that all pathogenic strains (100%) could form a biofilm, as previously documented by Chaves et al. (2020), who reported that 67% of poultry meat strains of *A. butzleri* are biofilm producers [32]. Biofilm formation plays an important role in meat contamination in slaughterhouses, considering that *A. butzleri* are rare in fecal samples from health chickens, and the contamination of meat is frequently associated with the horizontal transference of pathogens by contaminated surfaces in the poultry industry [16,33].

AB165 and AB170 also presented adhesion to HeLa cells after 3 hours of infection (Figure 1A). Cell adherence and host colonization have been associated with the *cadF* and *cj1349* genes in campylobacter-like microorganisms [33]. An in vitro study with two human intestinal cell lines (the mucus-producing HT-29-MTX and HT29 Caco-2 cells) demonstrated a high capacity of *A. butzleri* to colonize and adhere to HT29-MTX cells. Moreover, after 24 h of infection, *A. butzleri* crossed the Caco-2 epithelial barrier [34]. 

Attachment to the surface of epithelial cells and intestinal invasion are the first steps of gastrointestinal diseases, but toxin production also represents a step toward pathogenicity, due to the intense tissue damage, the occurrence of inflammatory reactions, and the increased risk of sepsis it causes. In our study, all strains were cytotoxic and induced cyto-distending and vacuolating effects in Vero cells (Figure 1B). 

In order to better understand the pathogenicity of *A. butzleri*, we performed the inoculation of virulent strains in birds and mammals. The in vivo inoculation in SPF birds showed that the clinical signs were transient, and, despite the high dose, the 11-week-old chicks presented short-term inflammatory enteritis (Figure 3) with mild diarrhea (Figure 2A) between the third and the fourth days after the inoculation, recovering after this period. The fecal excretion of the agent was also limited to 2–4 days after infection. This clinical picture is compatible with the low frequency of *Aliarcobacter* isolation from intestinal content in broilers [35]. 

According to Ho et al. (2008), the prevalence of *A. butzleri* in fecal samples of chickens is low as a result of the avian body temperature (41 °C), as the strains grow at 18–37 °C. The authors also indicate that the pathogen may prefer the ileum over than anaerobic environmental of the cecum [36]. Here, we highlighted the change of color of the fecal cecum content four days after the inoculation of the AB170 strain (Figure 2B). New studies are necessary to investigate the patterns of susceptibility variations related to chicken age and infective doses. Here, we did not investigate crop colonization, but we believe that the colonization of the crop is very important, as it can influence excretion and the contamination of slaughterhouses during evisceration. 

Although the infection in the chickens resulted in a mild clinical picture, the inoculation in a mammal model revealed a severe inflammatory and hemorrhagic illness (Figure 4A and Figure 5A). Ultrastructural microscopy revealed a severe tissue injury (Figure 6A), with villi destruction and the presence of bacilli in the extracellular matrix. Previous in vivo *Aliarcobacter* investigations were frequently based on models of rats, pigs, and zebrafish (*Danio rerio*) [37]. These studies evidenced the presence of an inflammatory disease, necrosis of organs, intestinal fluid accumulation, and risk of invasion and sepsis. Here, we used the rabbit ileal loop model, that is frequently employed in pathogenicity studies about diarrheagenic *Escherichia coli*. Our results confirmed the occurrence of hemorrhagic enteritis, compatible with the more severe pathology of bloody diarrhea in humans, reported in the literature [38]. In addition, we believe that the mammal model could be useful in next studies about *A. butzleri*. 

## 4. Materials and Methods

### 4.1. Bacterial Isolates

In the present study, a total of 27 strains of *A. butzleri* (with virulence factors based on previous PCR screening) were selected for antimicrobial susceptibility profiling. These strains were obtained in a previous study from 231 samples of chicken meat collected from municipal markets and slaughterhouses in São Paulo state, Brazil [6]. In addition, the strains AB165, AB167, and AB170 were subjected to pathogenicity tests in vitro and in vivo. The strains were PCR-positive for the *cia*B, *aj*1349, *hec*A, *hec*B, *hec*F, *irg*A, *mvi*N, *cad*F, *pld*A, and *tly*A genes. Isolation was carried out on JM selective agar [39] under aerobic conditions for 48 to 72 h at 30 °C. Species identification and detection of virulence genes were accomplished by polymerase chain reaction, as previously described [18,40,41,42]. 

### 4.2. Determination of MIC Values 

The broth microdilution technique was employed to determine the MIC values according to the protocol described by the Clinical and Laboratory Standards Institute [43], utilizing an interpretive standard for *Campylobacter* spp. [1]. The TREK Diagnostic System (ThermoFisher Scientific, Waltham, MA, USA) *Campylobacter* Sensititre^®^ MIC Plates employed in the assays contained the following panel of antimicrobials: azithromycin, ciprofloxacin, erythromycin, gentamicin, tetracycline, florfenicol, nalidixic acid, telithromycin, and clindamycin. 

The inoculums (1–2 × 10^8^ CFU/mL) were prepared with pure overnight cultures (3–4 colonies) suspended in 4 mL of sterile saline, standardized in 0.5 MacFarland. The plates were inoculated with 100 µL per well, using a multichannel pipette. The plates were sealed and incubated at 36 °C in 10% CO_2_ for 48 h. Quality test controls were performed using the *E. coli* strain ATCC 25922 and the *Staphylococcus aureus* strain ATCC 29213. 

The values of MIC_50_ and MIC_90_ were determined according to the definitions of Schwarz et al. [44], while BioNumerics version 7.6 software (Applied Maths, Saint-Martens-Latem, Belgium) was used to analyze the data and to generate a similarity matrix of antimicrobial resistance profiles. A bacterium was considered MDR when it presented resistance to at least one antimicrobial of three or more distinct classes [45]. 

### 4.3. Patogenicity In Vitro Tests 

#### 4.3.1. Biofilm

Biofilm formation was evaluated in triplicate by the crystal violet technique [46]. Overnight cultures were diluted to an optical density (OD) at 620 nm of 0.20 (~109 CFU/mL) and 0.02 (~108 CFU/mL), and 100 μL was inoculated into 96-well polystyrene plates, which were incubated for 48 h at 37°C, in microaerophile conditions. After the incubation, 25 µL of a 1% crystal violet (CV) solution in 100% ethanol was added to the wells, which were additionally incubated at room temperature for 15 min. The wells were rinsed five times with distilled water. Biofilm formation was quantified by dissolving the remaining CV with a solution composed of 30% methanol and 10% acetic acid and measuring the absorbance at 570 nm. The biofilm formation index (BIF) was calculated based on the optical density (OD) of attached and free bacteria, and biofilm formation was categorized into four categories: strong (≥1.10), moderate (0.70 to 1.09), weak (0.35 to 0.69), and none (<0.35) [47]. 

#### 4.3.2. Bacterial Adherence and Toxicity

HeLa cells were purchased from the Adolfo Lutz Institute (São Paulo, Brazil) and were cultured in Dulbecco’s Modified Eagle Medium (DMEM) (GIBCO, Carlsbad, CA, USA), supplemented with 10% fetal bovine serum (FBS) (GIBCO, USA), 1% non-essential amino acids (GIBCO, USA), and penicillin–streptomycin (10,000 U/mL) (GIBCO, USA), in a 5% CO_2_ incubator. The adherent cells were subcultured every 2–3 days by treatment with a trypsin-EDTA (0.5%) solution (GIBCO, USA). For the adherence test, HeLa cells were subcultured in 24-well plates (10^5^ cells/well) and incubated for 48 h in a 5% CO_2_ incubator.

The adherence test was performed as described by Vieira et al. (2001) [48]. The bacteria were cultured in Luria Bertani broth (Difco, USA) under aerobic conditions for 18 h at 37 °C. For the adherence assay, the bacteria (1 × 10^8^ CFU/well) were added to HeLa cells in 24-well plates, the medium was replaced with DMEM without antibiotics, and a 2% mannose solution was added, followed by incubation for 3 h at 37 °C. Each well was then washed three times with phosphate-buffered saline (PBS) to remove the non-bound bacteria, and DMEM was replaced. After 3 h of incubation in the same conditions, the cells were washed with PBS and fixed with methanol for 1 hour.

To analyze the bacterial adherence to Hela cells, cell staining was performed using May–Grünwald–Giemsa staining. Briefly, the cells were immersed in the May–Grünwald solution for 20 min, then transferred to a Giemsa solution for 5 min and washed three times with distilled water. The cells were visualized using a microscope (Nikon Eclipse E2000). For the adherence pattern control, the following strains were used: the EPEC prototype strain E2348/69 for localized adherence, the DAEC prototype strain C1845 for diffuse adherence, the EAEC prototype strain 042 for aggregative adherence.

The cytotoxicity assay in Vero cells (Monkey Kidney) was performed in triplicates as described by Martins et al. (2015) [49]. The strains were cultured in Luria Bertani broth (LB) (Difco—BBL, Detroit, MI, USA) at 37 °C for 18 h, in the presence of 5 ng/mL of ciprofloxacin (Sigma-Aldrich, St. Louis, MO, USA). The supernatants were obtained after centrifugation at 8800× *g* for 30 min and were filtered using a 0.22 Millipore filter. The test was conducted after the inoculation of 50 µL of supernatant into the microplate wells containing a Vero cells monolayer. *E. coli* O157:H7 (EDL933) and *E. coli* DH5α supernatants were used as positive and negative controls, respectively. 

### 4.4. Patogenicity In Vivo Tests

#### 4.4.1. Experimental Inoculation of Chickens

A total of 24 specific pathogen-free chickens (11 weeks of age) were grouped, with 6 birds per cage. Three groups were inoculated with 0.1 mL of *A. butzleri* culture (1.0 × 10^9^ UFC/mL) by gavage (Day 0), and one group was kept as the negative control (non-inoculated). One bird was euthanized per day (day 1 to day 6), for the observation of gross lesions and to collect tissues for histopathology. Fecal samples were collected daily for 7 days. The fecal samples were diluted 1:9 in selective enrichment broth as described by Johnson, Murano (1999) and incubated in anaerobiosis at 30 °C for 48 h. Then, 10 μL of cultured broth was placed on a sterile cellulose membrane (0.45 μm) on Johnson and Murano agar. After one hour, the filters were removed, and the broth was seeded and incubated at 30 °C for 48–72 h. 

#### 4.4.2. Rabbit Ileal Loop 

One New Zealand white rabbit (female, 1.9 Kg) was subjected to laparotomy after inhalation anesthesia as described by Gioia-Di Chiacchio et al. (2018) [50]. The ileum was rinsed with sterile saline, and intestinal loops of 6 cm in length were ligated and separated by 3 cm inter-loops. These loops were inoculated with 1 mL of each strain (1 × 10^6^ CFU/mL), previously cultured in BHI plus 0.1% glucose, and incubated at 30 °C for 18 h at 200 rpm. Sterile PBS was inoculated as a negative control. 

After 12 h, the animal was humanely euthanized for a post-mortem examination. Fragments of 0.5 mm of ileum tissue were collected and fixed in 10% buffered formalin and included in paraffin blocks for histopathology examination. The tissues stained with hematoxylin/eosin were examined by light microscopy (Eclipse NiU Nikon, with the camera DS-U3, Software Ni Elements; Nikon Corporation, Tokyo, Japan).

Scanning Electron Microscopy (SEM) was employed for the ultrastructural study, after fixation of 1 mm of tissue with 2.5% glutaraldehyde (*v*/*v*) in 0.1 M phosphate buffer (pH 7.2 at 0 °C). After fixation, fragments were rinsed with 0.1 M sodium cacodylate buffer, followed by 1% osmium tetroxide (OsO_4_) (*v*/*v*) and ethanol dehydrated solutions. The tissues were dried using the critical point method, mounted onto SEM stubs sputter-coated with gold, and examined using a Quanta 250 scanning electron microscope (FEI Company, Hillsboro, OR, USA) at 12.5 kV and working distance of 7 mm. 

## 5. Conclusions

Poultry meat is a clearly underrated reservoir of *Aliarcobacter* strains resistant to fluoroquinolone, macrolide, and tetracycline. The resistance profile and pathogenicity of *A. butzleri* isolated from Brazilian poultry meat reveal a public health risk. During 2022, Brazil exported 4.822 million tons of poultry meat, but there is no regulation about the presence of *Aliarcobacter* spp. The data obtained in this study reinforce the need to improve the diagnostics and surveillance, as well as the adoption of preventive actions in the Brazilian poultry industry.

## Figures and Tables

**Table 1:** Resistance profiles of 27 strains of *Aliarcobacter butzleri* isolated from commercial poultry meat from São Paulo city, Brazil.

Resistant Profiles		Number of Strains
R1	Susceptible strains	5
R2	CLIN	2
R3	NAL/CIP	1
R4	NAL/CIP/CLIN	1
R5	FLOR/CLIN	3
R6	NAL/FLOR/CLIN	11
R7	TET/NAL/FLOR/CLIN	1
R8	NAL/AZI/CLIN	1
R9	NAL/AZI/FLOR/CLIN	1
R10	AZI/ERI/TELI/FLOR/CLIN	1

CLIN—clindamycin; NAL—nalidixic acid; CIP—ciprofloxacin; FLOR—florfenicol; TELI—telithromycin; TET—tetracycline; AZI—azithromycin; ERI—erythromycin.

## Data Availability

The data is available at https://www.teses.usp.br/teses/disponiveis/10/10133/tde-09042020-103046/pt-br.php (accessed on 26 December 2022).

## Supplementary Materials

The following are available online at https://www.mdpi.com/article/10.3390/antibiotics12020282/s1, Table S1: Minimum inhibitory concentration (MIC) values of antimicrobials assessed against 27 strains of *Aliarcobacter butzleri* isolated from commercial chicken meat from São Paulo, Brazil.
