# Supplementary material for: Antimicrobial Resistance and Pathogenicity of Aliarcobacter butzleri Isolated from Poultry Meat
Source: Antibiotics (Basel). 2023 Feb 1;12(2):282. doi: 10.3390/antibiotics12020282 (PMC9952011; doi:10.3390/antibiotics12020282)
Supplement: Supplementary file 1 [file antibiotics-12-00282-s001.zip › antibiotics-2154409-supplementary.pdf]

antibiotics

MDPI

Table S1. Minimum inhibitory concentration (MIC) values of antimicrobials assessed against 27 strains of *Alarcobacter butzleri* isolated from commercial chicken meat from São Paulo, Brazil.

Antimicrobial	Number of strains according to MIC (μg/mL)														MIC_{50}	MIC_{90}	%R
	≤0.015	0.03	0.06	0.12	0.25	0.5	1	2	4	8	16	32	64	>64			
Azithromycin	0	0	0	0	0	8	8	4	4	0	**1**	**2**	0	0	1.0	16	11.1
Ciprofloxacin	0	0	14	6	3	2	0	0	0	0	**1**	**1**	0	0	0.06	0.5	7.4
Erythromycin		0	0	0	0	0	2	13	10	1	0	**1**	0	0	2	4	3.7
Gentamicin				1	25	1	0	0	0	0	0	0	0	0	0.25	0.25	0
Tetracycline			0	0	0	0	5	13	8	0	**1**	0	0	0	2	4	3.7
Florfenicol		0	0	0	0	0	0	0	10	**1**	**6**	**10**	0	0	16	32	62.9
Nalidixic acid									0	2	3	6	**16**	0	64	64	59.2
Telithromycin	0	0	0	0	0	0	7	18	1	**1**	0	0	0	0	2	2	3.7
Clindamycin		0	0	0	0	0	0	1	5	**5**	**8**	**8**	0	0	16	32	77.2

*Gray cells are untested values; the sidebar indicates the breakpoints. Resistant strains were in bold. MIC_{50} and MIC_{90} values are those that inhibit 50% and 90% of tested strains. %R = % of resistant strains

1
